# Is Oxidative Stress in Mice Brain Regions Diminished by 2-[(2,6-Dichlorobenzylidene)amino]-5,6-dihydro-4*H*-cyclopenta[*b*]thiophene-3-carbonitrile?

**DOI:** 10.1155/2013/194192

**Published:** 2013-03-14

**Authors:** A. C. Fortes, A. A. C. Almeida, G. A. L. Oliveira, P. S. Santos, W. De Lucca Junior, F. J. B. Mendonça Junior, R. M. Freitas, J. L. Soares-Sobrinho, M. F. R. Soares

**Affiliations:** ^1^Postgraduate Program in Pharmaceutical Sciences, Federal University of PI, 64.049-550 Teresina, Piauí, Brazil; ^2^Department of Pharmacy, Federal University of Piaui, 64.049-550 Teresina, PI, Brazil; ^3^Federal University of Sergipe, Center for Biological and Health Sciences, Department of Morphology, 49.100-000 São Cristovão, SE, Brazil; ^4^Laboratory of Synthesis and Vectorization of Molecules, State University of Paraiba, 58.020-540 João Pessoa, PB, Brazil; ^5^Department of Pharmaceutical Sciences, Federal University of Pernambuco, 50740-520 Recife, PE, Brazil

## Abstract

2-[(2,6-Dichlorobenzylidene)amino]-5,6-dihydro-4*H*-cyclopenta[*b*]thiophene-3-carbonitrile, 5TIO1, is a new 2-aminothiophene derivative with promising pharmacological activities. The aim of this study was to evaluate its antioxidant activity in different areas of mice central nervous system. Male Swiss adult mice were intraperitoneally treated with Tween 80 dissolved in 0.9% saline (control group) and 5TIO1 (0.1, 1, and 10 mg kg^−1^). Brain homogenates—hippocampus, striatum, frontal cortex, and cerebellum—were obtained after 24 h of observation. Superoxide dismutase and catalase activities, lipid peroxidation and nitrite content were measured using spectrophotometrical methods. To clarify the 5TIO1's mechanism on oxidative stress, western blot analysis of superoxide dismutase and catalase was also performed. 5TIO1 decreased lipid peroxidation and nitrite content in all brain areas and increased the antioxidant enzymatic activities, specially, in cerebellum. The data of Western blot analysis did not demonstrate evidence of the upregulation of these enzymes after the administration of this compound. Our findings strongly support that 5TIO1 can protect the brain against neuronal damages regularly observed during neuropathologies.

## 1. Introduction

Human cells are constantly exposed to oxidants [1]. There is indisputable evidence that oxidative stress is involved in the pathogenesis of many complex human diseases, including diabetes; cardiovascular, neural, and psychiatric disorders; and in cancer progression [2–4]. These diseases have been associated with alterations in reactive oxygen species (ROSs) [5], reactive nitrogen species (RNSs), and nitric oxide (NO) [6].

Oxidative stress has been a common pathogenic mechanism underlying many major psychiatric disorders, such as anxiety, due to the intrinsic oxidative vulnerability of the brain [7]. Growing evidences have suggested correlation between the imbalance of antioxidant defense mechanism and anxiety-like behavior [8–14]. Therefore, the role and the beneficial effects of antioxidants against various disorders and diseases induced by oxidative stress have received much attention [15], and it becomes important to develop drugs that can possibly exert neuroprotective actions [16, 17].

Among the numerous antioxidants available to cells, sulfur compounds (including cysteine, methionine) and glutathione and their derivatives have been widely studied due to their antioxidant properties [18]. Thereby, sulfur containing heterocycles paved the way for the active research in the pharmaceutical chemistry [19]. Currently, thiophene derivatives have attracted the interest of the pharmaceutical industry due to its broad pharmacological spectrum, specially, as anxiolytic [20] and antioxidant agents [21].

**Figure 1:**
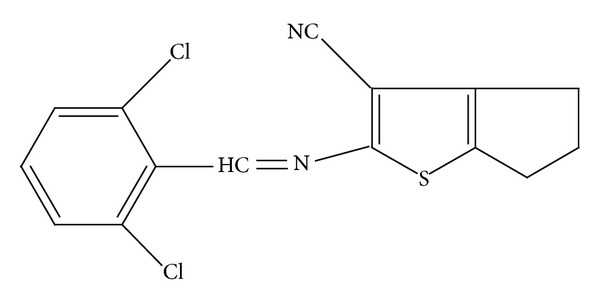
Chemical structure of 5TIO1.

In this context, the (2-[(2,6-dichlorobenzylidene)amino]-5,6-dihydro-4*H*-cyclopenta [*b*]thiophene-3-carbonitrile), also called 5TIO1 (Figure 1), is a new 2-aminothiophene derivative synthesized via two-step reaction, starting by the Gewald reaction, followed by the condensation with 2,6-dichlorobenzaldehyde, according to previous procedures [22]. 5TIO1 showed anxiolytic-like effect in plus-maze, and light/dark box tests did not change locomotor and coordination activities in open field and rotarod tests, respectively [23].

The aim of this study was to evaluate the antioxidant activity of 5TIO1 in different areas of the central nervous system (CNS) of adult mice by determination of lipid peroxidation level, nitrite content and enzymatic activities of catalase (CAT), and superoxide dismutase (Mn-SOD). To clarify 5TIO1's mechanism on oxidative stress, western blot analysis of Mn-SOD and CAT was also performed in mice brain homogenates 24 h of observation.

## 2. Materials and Methods

### 2.1. Animals and Experimental Procedures

Male Swiss adult mice (25–30 g) were used. All animals were maintained at a controlled temperature (25 ± 2°C) under 12 h dark/light cycle with free access to water and food. All experiments were performed according to the Guide for the Care and Use of Laboratory Animals, from the US Department of Health and Human Services, Washington DC, 1985. This protocol was previously approved by the Animal's Ethic Committee at Federal University of Piaui (n^o^ 031/12).

5TIO1 was from Laboratory of Synthesis and Vectorization of Molecules of the State University of Paraiba. It is a yellow crystal, produced in 89% of yield, melting point 159-160°C, *R*_*f*_ 0.54 (n-Hex./AcOEt. 8.5 : 1.5). It was emulsified with 0.05% Tween 80 (Sigma Chem. Co., St. Louis, Mo, USA), dissolved in 0.9% saline (vehicle), and intraperitoneally administered at doses of 0.1, 1 and, 10 mg kg^−1^ (*n* = 7 per group). Control animals received 0.25 mL of 0.05% Tween 80 dissolved in 0.9% saline (*n* = 7).

For neurochemical assays, both 5TIO1 and control groups (*n* = 7) were killed by decapitation 24 h after treatment. Their brains were dissected on ice to remove hippocampus, striatum, frontal cortex, and cerebellum for determination of lipid peroxidation levels, nitrite content, and superoxide dismutase and catalase activities.

Drug dosages of 5TIO1 were determined from dose-response studies (data not shown) and from observation of dose currently used in animal's studies [23–25]. The doses of 5TIO1 used (0.1, 1, and 10 mg/kg) in the present study are not equivalent to those used by humans because rats have different metabolic rates.

### 2.2. Determination of Lipid Peroxidation and Nitrite Content

In all experimental procedures, 10% (w/v) homogenates of each brain's area were prepared for both groups. Lipid peroxidation in the 5TIO1 groups (*n* = 7 per group) and control animals (*n* = 7) was analyzed by measuring thiobarbituric acid reacting substances (TBARSs) in homogenates, as previously described by Draper and Hadley [26]. Briefly, the samples were mixed with 1 mL 10% trichloroacetic acid and 1 mL 0.67% thiobarbituric acid. Then, they were heated in boiling water bath for 15 mins, and then butanol (2 : 1, v/v) was added to the solution. After centrifugation (800 g, 5 mins), TBARSs were determined from the absorbance at 535 nm. The results were expressed in mmol min^−1^ *μ*g protein^−1^. To determine nitrite content of the control mice (*n* = 7) and 5TIO1 groups (*n* = 7 per group), 10% (w/v) homogenates was centrifuged (800 g, 10 mins). The supernatants were collected, and nitric oxide production was determined based on the Griess reaction [27]. Briefly, 100 *μ*L supernatant was incubated with 100 *μ*L of the Griess reagent (1% sulfanilamide in 1% H_3_PO_4_/0.1% *N*-(1-naphthyl)ethylenediamine dihydrochloride/1% H_3_PO_4_/distilled water, 1 : 1: 1 : 1, v/v/v/v) at room temperature for 10 mins. A_550_ was measured using a microplate reader. Nitrite concentration was determined from a standard nitrite curve generated using NaNO_2_. The results were expressed in *μ*M.

### 2.3. Determination of Superoxide Dismutase and Catalase Activities

Each brain area was ultrasonically homogenized in 1 mL 0.05 mM sodium phosphate buffer, pH 7.0. Protein concentration was measured by the Lowry's method [28]. The 10% homogenates were centrifuged (800 g, 20 mins), and the supernatants were used in superoxide dismutase and catalase assays. Superoxide dismutase activity in the 5TIO1 groups (*n* = 7 per group) and control animals (*n* = 7) was evaluated by using xanthine and xanthine oxidase to generate superoxide radicals [29]. They react with 2,4-iodophenyl-3,4-nitrophenol-5-phenyltetrazolium chloride to form a red formazan dye. The degree of inhibition of this reaction was measured to assess superoxide dismutase activity. The standard assay substrate mixture contained 3 mL xanthine (500 *μ*M), 7.44 mg cytochrome c, 3.0 mL KCN (200 *μ*M), and 3.0 mL EDTA (1 mM) in 18.0 mL 0.05 M sodium phosphate buffer, pH 7.0. The sample aliquot (20 *μ*L) was added to 975 *μ*L of the substrate mixture plus 5 *μ*L xanthine oxidase. After 1 min, the initial absorbance was recorded, and the timer was started. The final absorbance after 6 mins was recorded. The reaction was followed at 550 nm. Purified bovine erythrocyte superoxide dismutase (Randox Laboratories, Belfast, Northern Ireland, UK) was used under identical conditions to obtain a calibration curve showing the correlation between the inhibition percentage of formazan dye formation and superoxide dismutase activity and the results expressed as U mg protein^−1^.

Catalase activity in the 5TIO1 groups (*n* = 7 per group) and control animals (*n* = 7) was determined by the method that uses H_2_O_2_ to generate H_2_O and O_2_ [30]. The activity was measured by the degree of this reaction. The standard assay substrate mixture contained 0.30 mL H_2_O_2_ in 50 mL 0.05 mM sodium phosphate buffer, pH 7.0. The sample aliquot (20 *μ*L) was added to 980 *μ*L substrate mixture. The initial absorbance was recorded after 1 min, and the final absorbance after 6 mins. The reaction was followed at 230 nm. A standard curve was established using purified catalase (Sigma, St Louis, MO, USA) under identical conditions. All samples were diluted with 0.1 mmol L^−1^sodium phosphate buffer (pH 7.0) to provoke a 50% inhibition of the diluent rate (i.e., the uninhibited reaction). Results are expressed as mmol min^−1^ *μ*g protein^−1^ [30, 31].

### 2.4. Western Blot Analysis

In the immunoblotting assay (*n* = 4 per group), hippocampus, striatum, frontal cortex, and cerebellum homogenates were mixed with protein loading buffer (roti-Load 1, Carl Roth GmbH, Karlsruhe, Germany) according to manufacturer's procedure and placed in a heating bath (95°C) for 5 mins. Proteins were separated using SDS-PAGE (gradient gels from 5% to 25%). The protein amount loaded per lane was 10 Ag. After separation, the proteins were stained with Coomassie Brilliant Blue or transferred to nitrocellulose paper, and unspecific protein binding sites were blocked with blocking buffer (Chemicon International, Hofheim, Germany). The blots were incubated overnight with the primary antibodies against (1) catalase (polyclonal, UBI, Lake Placid, NY, USA, 1 : 1.500) and (2) Mn-SOD (polyclonal, Assayama, Japan, 1 : 800), followed by incubation with horseradish peroxidase-conjugated secondary antibody (goat antirabbit IgG+ peroxidase, Boehringer Mannheim GmbH, Germany, 1 : 1.000). Immunoreactivity was visualized using the ECL detection system (Amersham Pharmacia Biotech, Buckinghamshire, UK). The Western blots did not show the B-actin band, since in our results corrections were made for the variability in protein loading in the gels.

### 2.5. Statistical Analysis

Results are expressed as means ± SEM for the number of experiments, with all measurements performed in duplicate. The Student-Newman-Keuls test was used for multiple comparison of means of two groups of data. Differences were considered significant at *P* < 0.05. Differences in experimental groups were determined by two-tailed analysis of variance.

## 3. Results

### 3.1. Determination of Lipid Peroxidation and Nitrite Content

Tables 1 and 2 show the 5TIO1's effects in lipid peroxidation and nitrite content, respectively, in hippocampus, striatum, frontal cortex, and cerebellum of adult mice. Statistical analysis indicated a decrease superior to 87% in lipid peroxidation and nitrite content in all brain areas for all doses when compared to the control group (*P* < 0.05). There was no dose-dependent effect of 5TIO1, and its scavenging activity was, statistically, the same in all areas for both assays.

### 3.2. Determination of Superoxide Dismutase and Catalase Activities

Table 3 shows the 5TIO1's effects on catalase activity in hippocampus, striatum, frontal cortex, and cerebellum of adult mice. a significantly increase was found in catalase activity in hippocampus (136.43, 152.74, and 145.99% in groups treated with 5TIO1 0.1, 1.0, and 10.0 mg kg^−1^, resp.) and in cerebellum (36.61, 46.04, and 42.14% in groups treated with 5TIO1 0.1, 1.0, and 10.0 mg kg^−1^, resp.) in comparison with the control group (*P* < 0.05). In striatum, groups treated with 5TIO1 0.1 and 1.0 increased catalase levels in 5.37% and 36.72%, respectively; but only 5TIO1 10 group augmented significantly (57.72%). In frontal cortex, the catalase activity increased 32.87% and 51.17% in the 5TIO1 1.0 and 10.0 mg kg^−1^ groups, respectively, if compared to the control group, 5TIO1 0.1 and 5TIO1 1.0 groups (*P* < 0.05). Only 5TIO1 at the dose of 0.1 mg kg^−1^ showed a decrease of 31.54% in catalase activity in frontal cortex compared with control group (*P* < 0.05).

Table 4 shows the 5TIO1's effects on superoxide dismutase activity in hippocampus, striatum, frontal cortex, and cerebellum of adult mice. 5TIO1 at the dose of 0.1 mg kg^−1^ showed an increase in superoxide dismutase activity in hippocampus (58.93%), striatum (8.33%), frontal cortex (14.54%), and cerebellum (22.29%) compared to the control group (*P* < 0.05). 5TIO1 at the dose of 1 mg kg^−1^ increased 40.18% and 8.64% SOD activity in hippocampus and frontal cortex, respectively, (*P* < 0.05). On the other hand, in the dose of 10 mg kg^−1^ upregulation of this enzyme activity was not observed.

### 3.3. Western Blot Analysis

**Figure 2:**
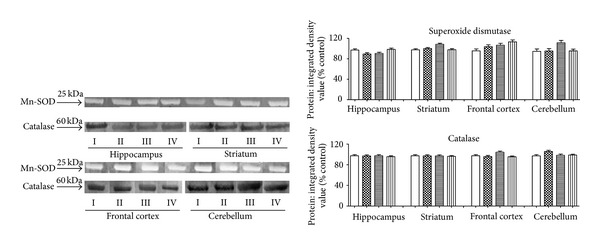
Superoxide dismutase and catalase proteins in hippocampus, striatum, frontal cortex, and cerebellum of mice treated with vehicle and 5TIO1. Male mice (25–30 g, 2 month-old) were intraperitoneally treated with 0.25 mL of 0.05% Tween 80 dissolved in 0.9% saline (vehicle) (*n* = 7, control group), and 5TIO1 groups with 2-[(2,6-dichlorobenzylidene)amino]-5,6-dihydro-4*H*-cyclopenta [*b*]thiophene-3-carbonitrile emulsified in vehicle (0.1, 1, and 10 mg/kg, *n* = 7, 5TIO1 groups). Animals were observed for 24 h and then killed. The protein amount per lane was 10 Ag. Legends: (I) control group; (II) 5TIO1 0.1 mg/kg group; (III) 5TIO1 1 mg/kg group; (IV) 5TIO1 10 mg/kg group. Data are expressed as means ± SD, converted to percentage of control (set at 100%) *n* = 4 experiments; Statistical analysis was by one-way ANOVA and *t*-Student-Neuman-Keuls test as *post hoc* test. There were no significant differences in protein expression of catalase after treatments, although there was a significant difference (*P* > 0.05).

In order to support the results obtained by Mn-SOD and CAT activities in the hippocampus, striatum, frontal cortex, and cerebellum, western blot analysis was also performed. After 24 h of the treatment with 5TIO1 (0.1, 1, and 10 mg kg^−1^) the total Mn-SOD and catalase activities did not change in comparison with control group. Likewise, 5TIO1 groups did not affect the Mn-SOD and catalase mRNA or protein levels, as tested by immunoblot analyses of hippocampus, striatum, frontal, and cerebellum homogenates of mice treated with 5TIO1 (Figure 2).

Expressed in relative arbitrary units, protein: integrated density value (% control) of Mn-SOD for 0.1, 1, and 10 mg kg^−1^ 5TIO1 groups were, respectively, 89.42 ± 2.48%, 90.58 ± 2.48% and 98.46 ± 2.48% in hippocampus; 100.2 ± 2.09%, 108.4 ± 2.09%, and 97.9 ± 2.09% in striatum; 103.7 ± 3.71%, 106.6 ± 3.71%, and 113.2 ± 3.71% in frontal cortex; and 95.36 ± 4.61%, 111.4 ± 4.61%, and 95.54 ± 3.42% in cerebellum. Analogously, protein levels of catalase for 0.1, 1, and 10 mg kg^−1^ 5TIO1 groups were, respectively, 97.07 ± 2.23%, 97.41 ± 2.23%, and 95.78 ± 1.66% in hippocampus; 97.23 ± 2.24%, 97.05 ± 2.24% and 96.27 ± 1.66% in striatum; 95.94 ± 2.18%, 104.4 ± 2.18% and 95.52 ± 1.62% in frontal cortex; and 105.7 ± 2.38%, 98.34 ± 2.38%, and 98.8 ± 1.77% in cerebellum (Figure 2).

## 4. Discussion

Oxidative stress, which represents a loss of balance in oxidation-reduction reactions, can dramatically alter neuronal function and has been related to anxiety [7, 10]. Our results observed neurochemical alterations after administration of an anxiolytic-like compound, 5TIO1 [23].

The brain is believed to be particularly vulnerable to oxidative stress due to neuronal membrane lipids, rich in highly polyunsaturated fatty acids, undergo rapid lipid peroxidation, and [32–34] with a few antioxidants defenses, such as low activity of catalase and superoxide enzymes [7]. Then, it is important to determine oxidative stress biomarkers which show a change in a biological molecule that has arisen from ROS and/or RNS attack. This concept is applied equally to products derived from lipids, proteins and antioxidant consumption [35].

Lipid peroxidation in a tissue is an index of irreversible biological damage of the cell membrane phospholipid, which, in turn, leads to inhibition of most of the sulfhydryl and some nonsulfhydryl enzymes [3, 36]. As TBARS levels are closely associated with lipid peroxidation [37], our findings demonstrated that 5TIO1 decreased lipid peroxidation in all brain areas, indicating its antioxidant protection.

The nitrite content is also a fundamental oxidative stress' biomarker, since high levels of nitric oxide and their oxidative derivatives, as peroxynitrite, can be toxic, playing an important role in neurodegenerative diseases [38–40]. In normal conditions, there is a steady-state balance between the production of nitric oxide and metabolites (nitrite and nitrate) and their destruction by antioxidant systems [41]. Our results show a decrease in nitrite formation after 5TIO1's administration, suggesting that this substance can prevent RNS formation.

Lipid peroxidation and nitrite content assays suggest that 5TIO1 can be an antioxidant by ROS and RNS scavenging, respectively. These results can be related with 5TIO1's physicochemical properties. Previous research identified sulfur-containing antioxidants as those with the most beneficial therapeutic ratio [42]. 5TIO1 contain a five-membered ring made up of one sulfur as heteroatom. Thus, it is a thiophene derivative. The sulfur atom in this five-membered ring acts as an electron donating heteroatom by contributing with two electrons to the aromatic ring [43]. So, the thiophene and benzene rings turn 5TIO1 into an electron-rich compound, suggesting that 5TIO1's antioxidant property could be linked with the free radicals' conversion into stable products through electron donation.

In normal conditions, there is a balance between oxidative and nitrosative stress and antioxidant actions. The harmful effect of free radicals to the organism induces several defense mechanisms [6, 36, 44]. One of them is the catalytic removal by factors such as catalase, superoxide dismutase, peroxidase, and thiol-specific antioxidants [45]. General protocols are described to measure the antioxidant enzyme activity of superoxide dismutase, catalase, and glutathione peroxidase. The superoxide dismutase converts superoxide radical into hydrogen peroxide and molecular oxygen, whereas the catalase, and peroxidase convert hydrogen peroxide into water. Western blots, activity gels, and activity assays are various methods used to determine protein and activity in both cells and tissues depending on the amount of protein required for each assay [46]. In order to understand 5TIO1's effect under CAT and Mn-SOD activity, we combined a sensitive protein quantification method—Lowry's method [47]—and western blot analysis, which have been the standard method for analyzing differences in protein levels from cells and tissues to compare experimental conditions [48].

Catalase is an enzyme that effectively reacts with H_2_O_2_ to produce water and molecular oxygen and with H donors (methanol, ethanol, formic acid, or phenols) with peroxidase activity. Catalase protects cells against H_2_O_2_ generated inside them. Although catalase is not essential to some cell types under normal conditions, it has an important role in the acquisition of tolerance to oxidative and nitrosative stress during the cellular adaptive response [6]. Superoxide dismutase is an important antioxidant enzyme that rapidly catalyzes the dismutation of superoxide anion (O^−∙^) and thus acts as a first line of defense. In the case of superoxide dismutase deficiency or increased superoxide production, it reacts with nitric oxide to produce peroxynitrite (ONOO^−^), which is a potent nitrosating agent that can cause direct damage to proteins, lipids, and DNA [49].

Our findings suggest higher catalase levels in cerebellum, frontal cortex, striatum, and hippocampus, indicating that this enzyme may exert a protective role in the first two brain regions more specifically over the other areas investigated in animals treated only with vehicle. Alper and coworkers [50] also observed that catalase activity is higher in cerebellum than the cerebral cortex of rodents. While superoxide dismutase activity is higher in cerebellum and striatum, indicating a better activity of this enzyme in these brain areas. So, the highest activity of both enzymes was observed in cerebellum in animals treated with vehicle, this result was similar to Fortunato and coworkers [51]. In our knowledge, this study presents, for the first time, results about oxidative stress in different mice brain areas, making it difficult to compare with other studies, since these data are unpublished for the areas investigated through the modulation of this new compound. So, this work motivates the interest in research and development of new drugs.

The superoxide dismutase activity was positively modulated by 5TIO1 at the dose of 0.1 mg kg^−1^ in all brain areas, specially, in hippocampus and cerebellum. However, no significant changes were observed with 10 mg kg^−1^. So, a dose-dependent process was not observed, suggesting that in higher doses of 5TIO1, there may be a saturation of this enzymatic activity. Furthermore, it should be noted that the 1 mg kg^−1^ increased superoxide activity only in hippocampus and frontal cortex, indicating that 5TIO1 exerts its possible antioxidant effect by Mn-SOD modulation in these areas. Like it was previously mentioned, these unpublished results will contribute significantly to fill the gaps of the causes and consequences of oxidative stress modulated by thiophenic compounds.

In our studies, the total Mn-SOD and catalase activities did not change after treatment with 5TIO1. Data from Western blot analysis did not demonstrate evidence for the upregulation of antioxidant enzymes (Mn-SOD and CAT) after treatment. In addition, data confirmed our hypothesis that occurred only an increase in the enzymatic activities studied, since there was no change in protein contents of Mn-SOD and catalase in treated mice (hippocampus, striatum, frontal cortex, and cerebellum) after 24 h of treatment with 5TIO1.

Moreover, the increased enzymatic activity observed in our study may be due to its allosteric regulation, since no studies have looked at our Western blot analyses that did not change in gene expression of these enzymes. However, the increased activity of the enzymes studied may also have been induced by covalent modification (phosphorylation). Thus, we suggest that during the establishment of oxidative stress in brains, the phosphorylation of these enzymes may play a central role to control neuronal functions involved in the establishment of anxiety disorders.

We clearly showed that 5TIO1 decreased the lipid peroxidation levels and nitrite content and increased the antioxidant enzymatic activities. In our knowledge, these effects of 5TIO1 on oxidative stress observed in brain have not been reported before. Thus, these findings might have important implications for understanding the mechanism of neurodegenerative diseases, promoting new advances in the development of selective, and targeted antiepileptic, antidepressant, and anxiolytic drugs. 5TIO1 can protect the brain against neuronal damages regularly observed during neuropathologies. Further investigation of 5TIO1 effects against necrosis, apoptosis, and/or autophagy observed in brains disorders are in progress to confirm its antioxidant and neuroprotective effects.

## Figures and Tables

**Table 1:** Determination of the lipid peroxidation levels in hippocampus, striatum, frontal cortex, and cerebellum of mice treated with 5TIO1 at doses 0.1, 1, and 10 mg kg^−1^.

Groups	TBARS (mmol min^−1^ *µ*g protein^−1^)			
	Hippocampus	Striatum	Frontal cortex	Cerebellum
Vehicle	1.36 ± 0.14	1.34 ± 0.06	1.54 ± 0.04	1.43 ± 0.06
5TIO1 0.1	0.13 ± 0.03^a^	0.13 ± 0.01^a^	0.12 ± 0.02^a^	0.13 ± 0.03^a^
5TIO1 1	0.16 ± 0.01^a^	0.16 ± 0.01^a^	0.15 ± 0.01^a^	0.18 ± 0.03^a,b^
5TIO1 10	0.15 ± 0.01^a^	0.15 ± 0.02^a^	0.15 ± 0.01^a^	0.17 ± 0.03^a,b^

Male mice (25–30 g, 2 month-old) were intraperitoneally treated with doses of 5TIO1 (0.1, 1, and 10 mg kg^−1^, *n* = 7, 5TIO1 groups) and the control animals with 0.05% Tween 80 dissolved in 0.9% saline (*n* = 7, Vehicle). Results are expressed as means ± SD for the number of animals shown inside in parenthesis. Differences in experimental groups were determined by two-tailed Analysis of Variance (ANOVA). ^a^ *P* < 0.05 compared to the control group (ANOVA and *t*-Student-Neuman-Keuls *as post hoc* test); ^b^ *P* < 0.05 compared to the 5TIO1 0.1 group (ANOVA and *t*-Student-Neuman-Keuls *as post hoc* test).

**Table 2:** Nitrite content in hippocampus, striatum, frontal cortex, and cerebellum of mice treated with 5TIO1 at doses 0.1, 1, and 10 mg kg^−1^.

Groups	Nitrite (*µ*M)			
	Hippocampus	Striatum	Frontal cortex	Cerebellum
Vehicle	95.86 ± 7.67	94.71 ± 3.35	76.57 ± 1.72	82.43 ± 1.99
5TIO1 0.1	5.11 ± 1.16^a^	6.00 ± 1.82^a^	6.49 ± 1.97^a^	6.93 ± 2.25^a^
5TIO1 1	6.36 ± 2.73^a^	4.98 ± 1.51^a^	6.19 ± 3.24^a^	4.81 ± 1.43^a^
5TIO1 10	7.14 ± 2.62^a^	9.04 ± 6.54^a^	8.50 ± 3.33^a^	9.04 ± 4.76^a^

Male mice (25–30 g, 2 month-old) were intraperitoneally treated with doses of 5TIO1 (0.1, 1, and 10 mg kg^−1^, *n* = 7, 5TIO1 groups) and the control animals with 0.05% Tween 80 dissolved in 0.9% saline (*n* = 7, Vehicle). Results are expressed as means ± SD for the number of animals shown inside in parenthesis. Differences in experimental groups were determined by two-tailed Analysis of Variance (ANOVA). ^a^ *P* < 0.05 compared to the control group (ANOVA and *t*-Student-Neuman-Keuls *as post hoc* test).

**Table 3:** Catalase activity in hippocampus, striatum, frontal cortex, and cerebellum of mice treated with 5TIO1 at doses 0.1, 1, and 10 mg kg^−1^.

Groups	Catalase (U *µ*g of protein^−1^)			
	Hippocampus	Striatum	Frontal cortex	Cerebellum
Vehicle	14.22 ± 1.54	19.35 ± 0.46	22.51 ± 0.37	24.61 ± 0.42
5TIO1 0.1	33.62 ± 2.52^a^	20.39 ± 2.79	15.41 ± 2.45^a^	33.62 ± 2.52^a^
5TIO1 1	35.94 ± 2.57^a^	26.36 ± 4.93	29.91 ± 5.54^a,b^	35.94 ± 2.57^a^
5TIO1 10	34.98 ± 3.24^a^	30.52 ± 7.12^a,b^	34.03 ± 3.32^a,b,c^	34.98 ± 3.24^a^

Male mice (25–30 g, 2 month-old) were intraperitoneally treated with doses of 5TIO1 (0.1, 1, and 10 mg kg^−1^, *n* = 7, 5TIO1 groups) and the control animals with 0.05% Tween 80 dissolved in 0.9% saline (*n* = 7, Vehicle). Results are expressed as means ± SD for the number of animals shown inside in parenthesis. Differences in experimental groups were determined by two-tailed Analysis of Variance (ANOVA). ^a^ *P* < 0.05 compared to the control group (ANOVA and *t*-Student-Neuman-Keuls *as post hoc* test); ^b^ *P* < 0.05 compared to the 5TIO1 0.1 group (ANOVA and *t*-Student-Neuman-Keuls *as post hoc* test); ^c^ *P* < 0.05 compared to the 5TIO1 1 group (ANOVA and *t*-Student-Neuman-Keuls *as post hoc* test).

**Table 4:** Superoxide dismutase activity in hippocampus, striatum, frontal cortex, and cerebellum of mice treated with 5TIO1 at doses 0.1, 1, and 10 mg kg^−1^.

Groups	Superoxide dismutase (U *µ*g of protein^−1^)			
	Hippocampus	Striatum	Frontal cortex	Cerebellum
Vehicle	2.24 ± 0.41	2.64 ± 0.49	2.20 ± 0.07	3.23 ± 0.35
5TIO1 0.1	3.56 ± 1.63^a^	2.86 ± 0.31^a^	2.52 ± 0.23^a^	3.95 ± 1.84^a^
5TIO1 1	3.14 ± 0.42^a^	2.54 ± 0.89	2.39 ± 0.44^a^	2.81 ± 1.96
5TIO1 10	2.16 ± 0.79	2.42 ± 0.38	2.17 ± 0.34	2.70 ± 1.34

Male mice (25–30 g, 2 month-old) were intraperitoneally treated with doses of 5TIO1 (0.1, 1, and 10 mg kg^−1^, *n* = 7, 5TIO1 groups) and the control animals with 0.05% Tween 80 dissolved in 0.9% saline (*n* = 7, Vehicle). Results are expressed as means ± SD for the number of animals shown inside in parenthesis. Differences in experimental groups were determined by two-tailed Analysis of Variance (ANOVA). ^a^ *P* < 0.05 compared to the control group (ANOVA and *t*-Student-Neuman-Keuls *as post hoc* test).
